# Efficacy of ultrasound-guided foam sclerotherapy in the healing of venous leg ulcers

**DOI:** 10.1016/j.jvsv.2025.102244

**Published:** 2025-04-08

**Authors:** Rashad A. Bishara, Ahmed Gaweesh, Ihab Nabil Hanna, Ahmed K. Allam, Mohamed R. Moabed, Sherif Essam, Wassila Taha, Alun H. Davies, Joseph Shalhoub

**Affiliations:** a Organization for Teaching Hospitals & Institutes of Egypt, Cairo, Egypt; b Vein Clinics Egypt, Cairo, Egypt; c Vascular Surgery Department, Alexandria University, Alexandria, Egypt; d iVein Clinic, Alexandria, Egypt; e Department of Vascular Surgery, National Institute for Diabetes and Endocrinology, Cairo, Egypt; f Department of General Surgery, Benha University Hospitals, Benha, Egypt; g Department of Vascular Surgery, Ain Shams University, Cairo, Egypt; h Section of Vascular Surgery, Department of Surgery & Cancer, Imperial College and Imperial Vascular Unit, Imperial College Healthcare NHS Trust, London, UK

**Keywords:** RCT, Venous leg ulcer, Foam sclerotherapy, Distal reflux

## Abstract

**Background:**

Ultrasound-guided foam sclerotherapy (UGFS) for treating the refluxing venous network beneath and in the vicinity of venous leg ulcers (VLUs) has been used widely.

**Objective:**

This trial aimed to assess the efficacy and safety of UGFS for treating VLUs (ISRCTN18090073).

**Methods:**

This study is a multicenter randomized controlled trial. Consenting eligible participants were randomized into two groups: group A received UGFS for the distal refluxing network of veins (eg, ulcer bed venous plexus), in addition to standard care, and group B received standard care alone. Standard care included compression therapy, wound care, ablation of superficial reflux, and recanalization of proximal deep venous obstruction when appropriate. Patients were followed weekly until complete ulcer healing was achieved. All participants will be followed for ≥12 months from the point of randomization to allow calculation of total ulcer-free days during the study period and assess for the recurrence of ulceration from the time of ulcer healing and quality of life measures. The primary outcome was the time to ulcer healing. Secondary outcomes were the rate of ulcer healing, Venous Clinical Severity Score, and patient-reported health-related quality of life.

**Results:**

The study was terminated early for efficacy at the planned interim analysis when one-half the number of patients had completed follow-up. A total of 71 patients, 6 with bilateral VLUs, were recruited. After accounting for dropouts and losses to follow-up, 65 VLUs were available for analysis. Both groups were similar at baseline regarding demographic factors, risk factors, history of deep vein thrombosis, previous venous interventions, and ulcer size. The time to complete ulcer healing was significantly shorter in group A, who received UGFS, as compared with group B, who did not receive UGFS (median, 35 days [interquartile range, 22 days] vs median, 56 days [interquartile range, 58 days]; *P* = .008). Additionally, more ulcers achieved complete healing within 3 months in group A compared with group B (28/29 [97%] vs 27/36 [75%]; *P* = .01). Multivariate regression analysis showed a significant effect of UGFS on healing time (*P* = .004). Group A showed a significantly more rapid reduction in ulcer size (*P* < .0001). There was a significant improvement in the Venous Clinical Severity Score after treatment in both groups A and B (*P* < .0001).

**Conclusions:**

Although both groups benefited from standard care for treating VLUs, the addition of UGFS improved treatment outcomes significantly. UGFS accelerated the healing process, resulting in a shorter time to complete ulcer healing and a higher rate of ulcers achieving complete healing within 3 months. These findings suggest that UGFS is a valuable adjunctive treatment for VLUs, enhancing the efficacy of standard care protocols.

**Article Highlights**

• **Type of Research:** Prospective randomized controlled trial
• **Key Findings:** We randomized 65 patients with open venous leg ulcers into two groups. The time to complete ulcer healing was significantly shorter in group A, who received ultrasound-guided foam sclerotherapy (UGFS), as compared with group B, who did not receive UGFS (median, 35 days [interquartile range, 22 days] vs median, 56 days [interquartile range, 58 days]; *P* = .008).
• **Take Home Message:** UGFS accelerated the healing process, resulting in a shorter time to complete ulcer healing and a higher rate of ulcers achieving complete healing within 3 months.

Venous leg ulcers (VLUs) are a significant health problem, affecting 0.06% to 1.69% of the population^1^ and causing considerable physical and psychological disability.^2^ VLUs are typically caused by reflux within the superficial venous system, reflux and/or obstruction of the deep venous system, or a combination of these factors. Additional contributors to the pathophysiology of VLUs include age, obesity, calf muscle pump impairment, genetic predisposition, and failure of microvenous valves.^3^^,^^4^

Compression therapy is the cornerstone of VLU treatment and has received a level 1A recommendation in the current Society for Vascular Surgery guidelines.^5^ EVRA (Early Venous Reflux Ablation) highlighted the positive impact of early ablation of superficial venous reflux on increasing the rate of complete ulcer healing and reducing the time to ulcer healing.^6^ Minimally invasive venous interventions have shown benefits in VLU healing.^7^ Despite these advances, there is no consensus on the adjunctive use of ultrasound-guided foam sclerotherapy (UGFS) for treating the refluxing venous network beneath and in the vicinity of a VLU. A recommendation has been made by the European Society for Vascular and Endovascular Surgery to ablate the subulcer venous plexus; however, it carries a level of evidence of C, indicating a lack of strong evidence.^8^

Previous cohort studies^9^, ^10^, ^11^, ^12^, ^13^ have reported the successful use of foam sclerotherapy to obliterate the refluxing network of veins in the vicinity of VLU. However, these studies were not randomized and, thus, their findings require further validation. A previous randomized study comparing compression alone vs compression with UGFS failed to recruit a sufficient number of patients for meaningful comparison.^14^ Another randomized study comparing ablation of axial reflux vs terminal interruption of the reflux source did not show a significant difference between the two groups.^15^

This study aimed to address this evidence gap regarding the effect of UGFS on VLU healing. Specifically, it sought to assess the efficacy and safety of UGFS for the treatment of VLU as an adjunct to standard care. The study protocol was registered with the ISRCTN registry (Trial ID: ISRCTN18090073).

## Methods

This study was designed as a multicenter, prospective, randomized, controlled trial to evaluate the efficacy and safety of UGFS for the treatment of VLU. The trial was conducted following the principles of the Declaration of Helsinki and was approved by the ethical committee of the General Organization of Teaching Hospitals and Institutes of Egypt.

Inclusion criteria for the trial were as follows:

• Patients with active VLUs;
• Evidence of superficial venous reflux or post-thrombotic deep venous reflux and/or obstruction via duplex ultrasound examination or venography;
• Presence of a refluxing network of veins in the vicinity of the ulcer (ulcer veins) with or without pathological incompetent perforators; and
• Age >18 years.

Exclusion criteria were as follows:

• Pregnant and lactating females;
• Peripheral arterial disease confirmed by an ankle-brachial pulse index of <0.8, or arterial duplex ultrasound examination;
• Absence of a refluxing network of veins in the vicinity of the ulcer;
• VLU duration >2 years;
• VLU size >20 cm in any dimension; and
• Inability to provide informed consent.

Individuals meeting the inclusion criteria were offered a participant information sheet. Written informed consent was obtained from each patient before randomization.

Eligible and consenting participants were assigned randomly to one of the two study groups using a digital randomization application. Randomization was performed using digital software which randomized patients on a 1:1 basis. There was no stratification.

• Group A (intervention) received UGFS for the distal refluxing network of veins in addition to standard care.
• Group B (control) received standard care alone.

The health care provider was then informed whether the patient should receive foam sclerotherapy or not, ensuring that the allocation process was concealed until assignment. Neither the health care providers nor the patients were blinded to the intervention after allocation.

Standard care for both groups included the following:

• Compression therapy provided by trained personnel using multilayer elastic compression, short-stretch compression, compression stockings, or adjustable compression wraps;
• Wound care with debridement and antibiotics, as needed;
• Ablation of superficial venous reflux via surgery or endovenous techniques, if applicable; and
• Recanalization of proximal deep venous obstruction when appropriate.

All ulcers were photographed. The ulcer area was measured in square centimeters by way of a standardized process using digital software. The VLU location was recorded.

Consenting individuals randomized to group A, in addition to receiving standard care as described, received UGFS by a standardized protocol. This involved identification of ulcer veins (as defined in the inclusion criteria) using a linear ultrasound probe (7-10 Mhz). Injection of polidocanol (0.5%-110%) mixed with gas in a 1:4 ratio to fill the refluxing ulcer veins. A total volume of 5 to 10 mL of foam per session, with up to two sessions permitted per limb if ulcer veins remained patent after 1 week of follow-up. Any associated incompetent perforators were treated by USGF as part of the treatment of the refluxing network of veins in the vicinity of the ulceration. After foam sclerotherapy, compression bandages were applied.

For individuals in both groups, ulcer dressing and reapplication of compression were performed on a weekly basis until the ulcer healed. Bandages were used during the active phase of the ulcer. Multilayer bandages were used, including elastic and short stretch inelastic bandages. After ulcer healing, grade II compression stockings or adjustable compression wraps were prescribed. After complete healing, patients will be followed up monthly by telephone to enquire about any ulcer recurrence and compliance with compression hosiery. At completion of the study, patients will be reviewed in person.

The study primary outcome was time to complete ulcer healing, assessed weekly until healing was achieved. Secondary outcomes were as follows:

• Rate of ulcer healing, measured by change in ulcer size during the study period;
• Complete ulcer healing at 3, 6, 9, and 12 months after randomization;
• Venous Clinical Severity Score^16^ before and after treatment, and at 12 months after randomization; and
• Quality-of-life assessment using the Short Form-12 (Arabic language version) before and after treatment, and at 12 months after randomization.

The sample size was calculated at 90% power with an alpha of 5%. If, as in EVRA, a 15% effect size was considered, this would make the healing time estimate for compression and intervention 64.1 ± 26.0 days; the sample size would be 112 VLUs. Allowing for a dropout rate of 10%, the target sample size was estimated to be 130 VLUs. An interim analysis was decided at 50% of the target sample size, at which point 65 VLUs would have completed follow-up. According to the a priori protocol, if the intervention group (group A) showed a significant difference compared with the group treated conservatively (group B), the study would be terminated early for evidence of efficacy.

Statistical analysis was performed using SPSS version 28 (IBM, Armonk, NY). Descriptive statistics summarized the demographic and clinical characteristics of the study population. Normality testing was performed, and non-normally distributed data was expressed as median and interquartile range (IQR). The Mann-Whitney *U* test was used to compare nonparametric data between groups. Kaplan-Meier analysis was used to compare the time to complete ulcer healing between the two groups. The significance level for all statistical tests was set at α = 0.05.

## Results

**Fig 1:**
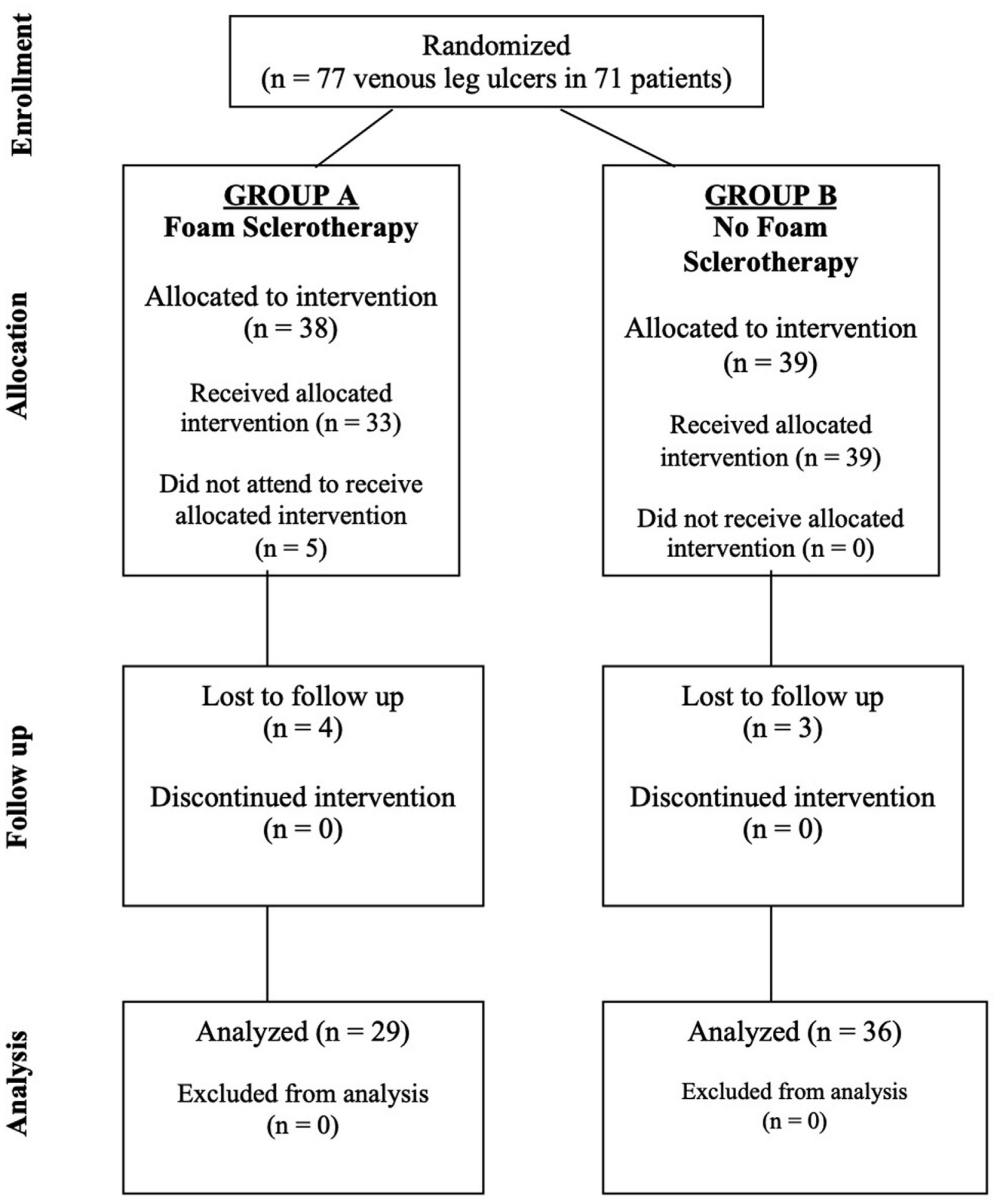
CONSORT diagram for the randomised controlled trial.

Recruitment took place over 24 months, from January 2022 to December 2023, at six centers throughout Egypt. Seventy-one patients with 77 VLUs (6 patients had bilateral ulcers), were randomized. Five patients with unilateral VLU did not attend for intervention after randomization, and seven patients with unilateral ulcers were lost to follow-up after the intervention. At total of 65 VLUs completed follow-up and were available for analysis: 29 in group A, who received UGFS for ulcer veins, and 36 in group B, who did not receive UGFS. The trial CONSORT diagram is shown in Fig 1. No major complications were reported owing to UGFS, including no anaphylaxis, neurological complications, visual symptoms, chest tightness, or superficial or deep vein thrombosis.

No significant differences were observed between the groups in terms of demographic factors, risk factors, history of deep vein thrombosis, previous venous interventions, or ulcer size. However, ulcer duration was significantly shorter in group A (median, 4 months; IQR, 4 months) compared with group B (median, 6 months; IQR, 4 months; *P* = .02). There was no significant difference between the two groups in terms of prerandomization duplex ultrasound findings (Table).

**Table:** Comparison of demographic factors, ulcer characteristics, history of deep vein thrombosis, history of venous interventions, risk factors, and prerandomization duplex ultrasound findings

	Group A, foam sclerotherapy (n = 29)	Group B, no foam sclerotherapy (n = 36)	*P* value
Male sex	20 (69)	24 (67)	.84
Age, years	46.97 ± 14.43	48.22 ± 14.90	.73
No. of ulcers	1.2 (83% had 1 ulcer)	1.39 (72% had 1 ulcer)	.60
Ulcer size, cm^2^	3.15 [3.75]	6 [9.66]	.10
Ulcer duration, months	4.0 [4.0]	6 [4.5]	**.02**
History of deep vein thrombosis	13 (45)	12 (33)	.34
Previous venous intervention	13 (45)	12 (33)	.89
Diabetes mellitus	3 (10)	3 (8)	.78
Body mass index (kg/m^2^)	29.1 [10.5]	34.7 [9.8]	.60
Duplex superficial GSV/SASV/AASV reflux	11 (38)	18 (50)	.23
Duplex superficial SSV reflux	10 (34)	12 (35)	.92
Duplex extra-axial varicose veins	11 (38)	15 (42)	.75
Duplex iliac vein obstruction	3 (10)	1 (3)	.20
Duplex infrainguinal PTS	14 (48)	12 (33)	.22
Duplex incompetent ulcer veins or incompetent pathological perforator	29 (100)	36 (100)	-

*AASV,* Anterior accessory saphenous vein; *GSV,* great saphenous vein; *PTS,* post-thrombotic syndrome; *SASV,* superficial accessory saphenous vein; *SSV,* small saphenous vein.

In group A, 83% had one ulcer; in group B, 72% had one ulcer (*P* = .6).

Values are number (%), mean ± standard deviation, or median [interquartile range].

Boldface entries indicate statistical significance.

Group A had 21 medial, 7 lateral, and 1 posterior VLUs, and group B had 29 medial, 5 lateral, and 2 posterior VLUs. There was no significant difference between the two groups in ulcer location (*P* = .4).

All patients in group A were treated with one session of UGFS, except one who had a second sessions. The Tessari method was used to make foam using either air (12 VLUs) or carbon dioxide (17 VLUs) with a ratio of liquid to gas 1:4. Interventions were performed within 2 weeks of randomization. All patients in group A had a duplex ultrasound examination 1 week after UGFS to check for obliteration of the venous plexus and to check for deep vein thrombosis.

The number of limbs that were treated by ablation of axial reflux was 10 of 29 (34%) in group A and 18 of 36 (50%) in group B (*P* = .2). The management of axial reflux was the same in both groups, with thermal ablation being performed for above-knee great saphenous vein reflux in both groups. None of the patients in group A had iliac vein stenting, and only one patient in group B had iliac vein stenting. All patients who required treatment for incompetent tributaries of the proximal leg were treated by foam sclerotherapy (six in each group); none was treated with phlebectomies.

### Primary outcome

**Fig 2:**
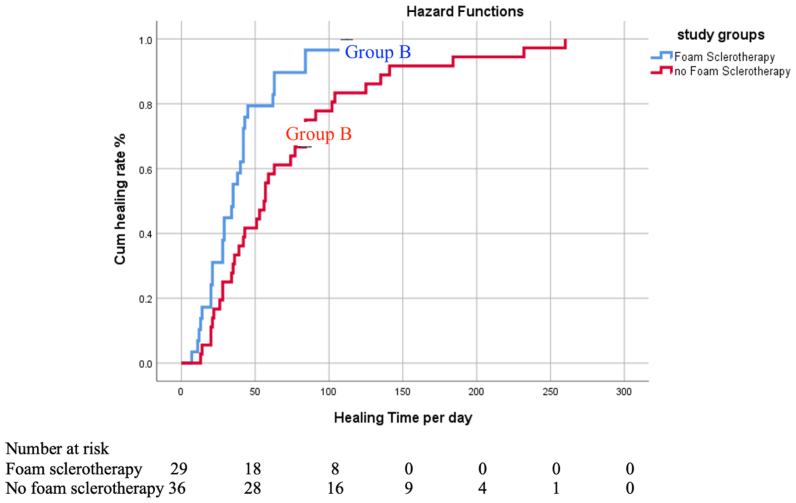
Kaplan-Meier curve for time to ulcer healing in the two different groups (*P* = .003).

The time to complete ulcer healing was significantly shorter in group A as compared with group B (median, 35 days [IQR, 22 days] vs median, 56 days [IQR, 58 days]; *P* = .008) (Fig 2 and Supplementary Fig 1, online only). According to the a priori protocol, the study was, therefore, terminated early for evidence of efficacy.

### Secondary outcomes

A greater number of ulcers in group A reached complete healing within 3 months compared with group B (28/29 [97%] vs 27/36 [75%]; *P* = .01). All patients in group A healed within 6 months, compared with 33 of 36 (92%) of group B. All patients in group B healed within 9 months.

Owing to the finding of a longer ulcer duration in the no foam sclerotherapy group (group B), post hoc analyses were performed to determine the impact of this baseline factor on the trial primary outcome. Kendall's tau and Spearman's rho correlations tests assessed correlations between variables and treatment groups. Multivariate regression analysis assessed the effect of variables on time to complete healing in the two groups. Ulcer duration was a confounder in the relationship between treatment groups and healing time, but it did not show a significant correlation or significant effect on the healing time (Supplementary Tables I and II, online only).

**Fig 3:**
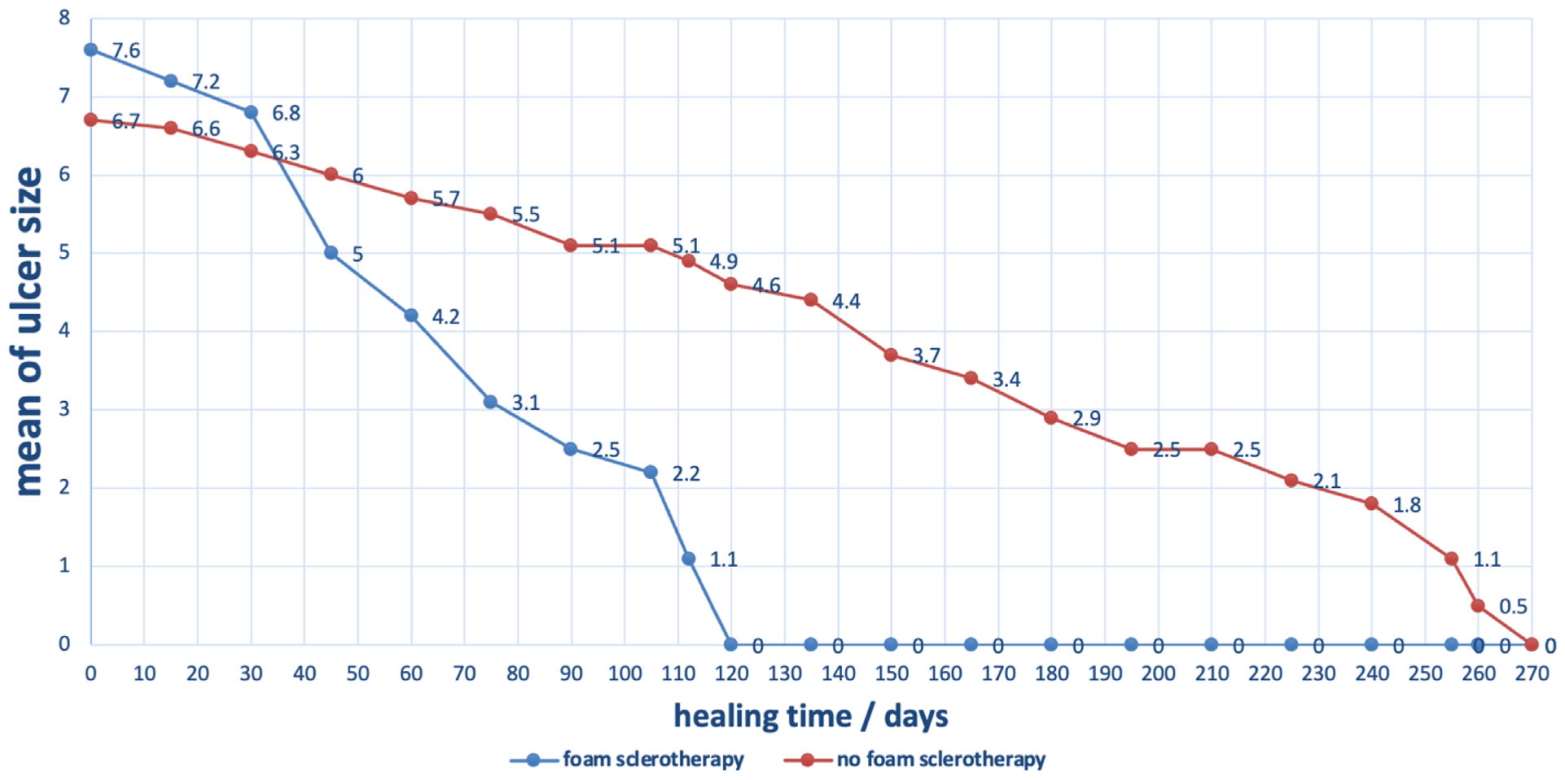
The two-line graphs depict the rate of ulcer healing for the two groups: one treated with foam sclerotherapy (group A) and one without (group B). The vertical axis represents the mean ulcer size in square centimeters, and the horizontal axis represents time since randomization in days. All ulcers were photographed. The ulcer area was measured in square centimeters by way of a standardized process using digital software. Multivariate analysis showed a significant difference between the two groups (*P* < .0001).

Group A, treated with UGFS, showed a more rapid and significant reduction in ulcer size as compared with group B. Multivariate analysis revealed a significant difference between the healing rates of the two groups (*P* < .0001) (Fig 3).

The revised Venous Clinical Severity Score showed significant improvements within both groups with treatment (*P* < .0001), although no significant differences were observed between the groups after treatment (Supplementary Fig 2, online only).

All participants will be followed for ≥12 months from the point of randomization to allow calculation of total ulcer-free days during the study period and assess for recurrence of ulceration from the time of ulcer healing, as well as quality of life measures. Because this follow-up has not been completed, these results will be the subject of a subsequent publication.

## Discussion

The key finding of this randomized trial is that UGFS of the ulcer veins significantly decreases healing time when used as an adjunct to standard of care for VLUs. This finding aligns with previous nonrandomized studies describing the beneficial effect of UGFS on abolishing distal venous reflux and improving VLU healing outcomes.^17^, ^18^, ^19^ The trial was terminated early for evidence of efficacy in accordance with the a priori study protocol.

Two previous randomized controlled trials addressed UGFS for ulcer veins. One was terminated owing to inadequate patient recruitment, and the second compared the obliteration of axial reflux with UGFS for obliterating terminal reflux, showing no difference between groups. In this study, patients with axial reflux received ablation in both groups, allowing a clearer evaluation of the effect of UGFS administered to veins in proximity to VLU.

UGFS of ulcer veins aims to obliterate the network of veins deep to and in the vicinity of the ulcer, including any incompetent perforating veins present. This technique is an augmentation of other techniques that target only incompetent perforators,^20^ as UGFS has the potential to obliterate the entire subulcer venous network, hypothesized to provide a more comprehensive treatment approach.

The Table shows that the two groups were equal in demographic factors, risk factors, and duplex findings, except for ulcer duration on presentation. Ulcer duration was significantly longer in group B. However, ulcer duration did not show significant correlation with healing time (Supplementary Table I, online only) and was found to be only a confounder on multiple regression analysis (Supplementary Table II, online only).

Fig 3 presents the rate of ulcer healing in both groups. At the beginning of the study, the mean ulcer size was larger in group A, 7.62 cm^2^, compared with group B, 6.70 cm^2^. Group A showed a rapid decrease of ulcer size within the first 2 months, decreasing to a mean of 4.2 cm^2^ by 60 days, and a steady decrease in ulcer size until all ulcers healed by the 120 days. Group B showed a slower decrease in ulcer size, which extended to 270 days until all ulcers healed. The difference in the ulcer healing rate was statistically significant (*P* < .0001).

By 3 months, almost all patients receiving UGFS had experienced ulcer healing. Those not receiving foam sclerotherapy saw healing extending over a longer period; many ulcers in this group remained unhealed, with some taking ≤9 months to heal completely.

Although patients in group A, who were treated with UGFS of the ulcer veins, showed a shorter healing time, both groups showed a relatively short healing time if compared with real-world VLU healing times^21^; this finding could be attributed to the strict adherence to the protocol, including the application of compression therapy, early ablation of superficial venous reflux, and recanalization of the proximal deep venous system occlusion when appropriate. The short healing time in both groups could also be attributed to the exclusion criteria, whereby ulcers that were present for >2 years in duration or >20 cm^2^ were excluded.

This study has some limitations, including the fact that participants and health care providers were not blinded to the intervention after randomization and the relatively small total number of patients recruited. The latter is in accordance with the trial protocol that indicated an interim analysis at 50% target completion of follow-up. Other limitations are the lack of standardization in the use of venoactive medications and lack of the data regarding the use of ulcer debridement and antibiotics.

## Conclusions

Both groups in this study seemed to benefit from the application of the standard of care for the treatment of VLU; however, the addition of UGFS significantly shortened the time to achieve complete ulcer healing.

## Author contributions

Conception and design: RB, AG, SE, WT, AD, JS

Analysis and interpretation: RB, AG, AD, JS

Data collection: RB, IH, AA, MM, WT

Writing the article: RB, AG, JS

Critical revision of the article: RB, AG, IH, AA, MM, SE, WT, AD, JS

Final approval of the article: RB, AG, IH, AA, MM, SE, WT, AD, JS

Statistical analysis: Not applicable

Obtained funding: Not applicable

Overall responsibility: RB

## Funding

None.

## Disclosures

None.

## References

[1] Probst S., Saini C., Gschwind G. (2023). Prevalence and incidence of venous leg ulcers-A systematic review and meta-analysis. Int Wound J.

[2] Herber O.R., Schnepp W., Rieger M.A. (2007). A systematic review on the impact of leg ulceration on patients' quality of life. Health Qual Life Outcomes.

[3] Raffetto J.D., Ligi D., Maniscalco R., Khalil R.A., Mannello F. (2020). Why venous leg ulcers have difficulty healing: overview on pathophysiology, clinical consequences, and treatment. J Clin Med.

[4] Vincent J.R., Jones G.T., Hill G.B., van Rij A.M. (2011). Failure of microvenous valves in small superficial veins is a key to the skin changes of venous insufficiency. J Vasc Surg.

[5] O'Donnell T.F., Passman M.A., Marston W.A. (2014 Aug). Society for Vascular surgery; American Venous Forum. management of venous leg ulcers: clinical practice guidelines of the Society for Vascular Surgery ® and the American Venous Forum. J Vasc Surg.

[6] Gohel M.S., Heatley F., Liu X., EVRA trial Investigators (2018). A randomized trial of early endovenous ablation in venous ulceration. N Engl J Med.

[7] Montminy M.L., Jayaraj A., Raju S. (2018). A systematic review of the efficacy and limitations of venous intervention in stasis ulceration. J Vasc Surg Venous Lymphat Disord.

[8] De Maeseneer M.G., Kakkos S.K., Aherne T. (2022). Editor's Choice - European Society for Vascular Surgery (ESVS) 2022 clinical practice guidelines on the management of chronic venous disease of the lower limbs. Eur J Vasc Endovasc Surg.

[9] Liu X., Zheng G., Ye B., Chen W., Xie H., Zhang T. (2019). Comparison of combined compression and surgery with high ligation-endovenous laser ablation-foam sclerotherapy with compression alone for active venous leg ulcers. Sci Rep.

[10] Zhu Y., Wu D., Sun D., Song K., Li J., Lin J. (2020). Ultrasound- and fluoroscopy-guided foam sclerotherapy for lower extremity venous ulcers. J Vasc Surg Venous Lymphat Disord.

[11] Cuffolo G., Hardy E., Perkins J., Hands L.J. (2019). The effects of foam sclerotherapy on ulcer healing: a single-centre prospective study. Ann R Coll Surg Engl.

[12] Grover G., Tanase A., Elstone A., Ashley S. (2016). Chronic venous leg ulcers: effects of foam sclerotherapy on healing and recurrence. Phlebology.

[13] Kamhawy A.H., Elbarbary A.H., Elhenidy M.A., Elwagih A.M.M. (2020). Periulcer foam sclerotherapy injection in chronic venous leg ulcers using near-infrared laser for vein visualization. Int J Low Extrem Wounds.

[14] O'Hare J.L., Earnshaw J.J. (2010). Randomised clinical trial of foam sclerotherapy for patients with a venous leg ulcer. Eur J Vasc Endovasc Surg.

[15] Keohane C.R., Westby D., Twyford M., Aherne T., Tawfick W., Walsh S.R. (2024). Axial ablation versus terminal interruption of the reflux source (AAVTIRS): a randomised controlled trial. Vasc Endovascular Surg.

[16] Vasquez M.A., Rabe E., McLafferty R.B. (2010). American Venous Forum Ad hoc outcomes working group. Revision of the venous clinical severity score: venous outcomes consensus statement: special communication of the American Venous Forum Ad hoc outcomes working group. J Vasc Surg.

[17] Joyce D.P., De Freitas S., Woo E.Y., Tang T.Y., Tubassam M., Walsh S.R. (2022). Ultrasound-guided foam sclerotherapy as a therapeutic modality in venous ulceration. Surgeon.

[18] Weber B., Marquart E., Deinsberger J., Tzaneva S., Böhler K. (2022). Comparative analysis of endovenous laser ablation versus ultrasound-guided foam sclerotherapy for the treatment of venous leg ulcers. Dermatol Ther.

[19] Pihlaja T., Kosunen E., Ohtonen P., Pokela M. (2024). Sub-ulcer foam sclerotherapy in patients with venous leg ulcer, analysis and technical aspects of 134 consecutive patients. Int J Low Extrem Wounds.

[20] Giannopoulos S., Rodriguez L., Chau M. (2022). A systematic review of the outcomes of percutaneous treatment modalities for pathologic saphenous and perforating veins. J Vasc Surg Venous Lymphat Disord.

[21] Goldschmidt E., Schafer K., Lurie F. (2021). A systematic review on the treatment of nonhealing venous ulcers following successful elimination of superficial venous reflux. J Vasc Surg Venous Lymphat Disord.

