# Supplementary material for: Efficacy of ultrasound-guided foam sclerotherapy in the healing of venous leg ulcers
Source: J Vasc Surg Venous Lymphat Disord. 2025 Apr 8;13(4):102244. doi: 10.1016/j.jvsv.2025.102244 (PMC12138539; doi:10.1016/j.jvsv.2025.102244)
Supplement: Supplementary Material [file mmc1.docx]

**Appendix**

**A Multi-Center Randomized Controlled Trial to Evaluate the Efficacy of Ultrasound-Guided Foam Sclerotherapy in Venous Leg Ulcers**

| Healing Time in Days | Spearman's rho | | Kendall's tau_b | |
| --- | --- | --- | --- | --- |
|  | Correlation Coefficient | Sig. (2-tailed) | Correlation Coefficient | Sig. (2-tailed) |
| Age Groups | 0.005 | 0.963 | 0.006 | 0.96 |
| Gender | -0.071 | 0.496 | -0.085 | 0.50 |
| Number of ulcers | 0.169 | 0.096 | 0.202 | 0.10 |
| Ulcer area | 0.241 | **0.005** | 0.349 | **0.004** |
| Ulcer duration in months | 0.139 | 0.119 | 0.197 | 0.11 |
| Weight in Kg | 0.260 | **0.003** | 0.367 | **0.003** |
| Height in meter | 0.142 | 0.107 | 0.196 | 0.11 |
| BMI | 0.140 | 0.105 | 0.213 | 0.08 |
| Ulcer depth | 0.031 | 0.768 | 0.03 | 0.77 |
| Ulcer location | 0.133 | 0.193 | 0.167 | 0.18 |
| History of DVT | 0.022 | 0.834 | 0.026 | 0.83 |
| DM | -0.111 | 0.286 | -0.133 | 0.29 |
| Myocardial impairment | -0.110 | 0.287 | -0.133 | 0.29 |

**Table I supplemental.** Correlation between healing time and all variables.

| Coefficients ^a^ | | | | | |
| --- | --- | --- | --- | --- | --- |
| Factors | | Unstandardized Coefficients | t | Sig. | Effect on healing time among the groups |
|  |  | B |  |  |  |
| 1 | (Constant) | **1.266** | **10.003** | **.000** | **Significance association** |
|  | study groups | **.235** | **3.031** | **.004** |  |
|  | study groups | .239 | 3.084 | .003 | Non |
|  | Age | -.003 | -1.140 | .259 |  |
| 2 | Study groups | .236 | 3.032 | .004 | Non |
|  | Gender | -.054 | -.652 | .517 |  |
| 3 | study groups | .232 | 2.937 | .005 | Non |
|  | Number of ulcers | .024 | .345 | .731 |  |
| 4 | study groups | .221 | 2.826 | .006 | Non |
|  | Ulcer area | .004 | 1.231 | .223 |  |
| 5 | study groups | .237 | 3.032 | .004 | Non |
|  | Ulcer depth | .076 | .566 | .574 |  |
| 6 | study groups | .229 | 2.924 | .005 | Non |
|  | Ulcer location | .054 | .780 | .439 |  |
| 7 | study groups | .213 | 2.619 | **.011** | **Confounder** |
|  | Ulcer duration in months | .007 | .929 | **.356** |  |
| 8 | study groups | .240 | 3.056 | .003 | Non |
|  | History of DVT | .043 | .537 | .593 |  |
| 9 | study groups | .233 | 2.993 | .004 | Non |
|  | DM | -.110 | -.821 | .415 |  |
| 10 | study groups | .260 | 3.332 | .001 | Non |
|  | Myocardial impairment | -.302 | -1.631 | .108 |  |
| 11 | study groups | .182 | 2.378 | **.021** | **Effect modifier** |
|  | Weight in Kg | .005 | 2.721 | **.008** |  |
| 12 | study groups | .230 | 3.044 | **.003** | **Effect modifier** |
|  | Height in meter | .685 | 2.071 | **.043** |  |
| 13 | study groups | .213 | 2.711 | .009 | Non |
|  | BMI | .007 | 1.395 | .168 |  |
| 14 | study groups | .226 | 2.872 | .006 | Non |
|  | Duplex Superficial GSV/SASV/ AASV Reflux | .062 | .786 | .435 |  |
| 15 | study groups | .236 | 3.035 | .004 | Non |
|  | Duplex Superficial SSV Reflux | .071 | .864 | .391 |  |
| 16 | study groups | .234 | 2.992 | .004 | Non |
|  | Duplex Extra-axial varicose veins | .038 | .484 | .630 |  |
| 17 | study groups | .229 | 2.899 | .005 | Non |
|  | Duplex iliac v. obstruction | -.079 | -.485 | .630 |  |
| 18 | study groups | .235 | 2.968 | .004 | Non |
|  | Duplex infra-inguinal PTS | -.002 | -.028 | .977 |  |
| 19 | study groups | .257 | 3.264 | .002 | Non |
|  | Duplex incompetent ulcer veins | .256 | 1.378 | .173 |  |
| 20 | study groups | .235 | 3.025 | .004 | Non |
|  | Duplex incompetent pathologic perforator | -.066 | -.782 | .437 |  |
| 21 | study groups | .240 | 3.045 | .003 | **Non** |
|  | **Ablation of superficial axial reflux** | **8.10** | **0.62** | **0.536** |  |

**Table II supplemental.** Results of multivariate regression analysis. The dependent variable was healing time.

**Figure I supplemental.** Time to complete ulcer healing was significantly shorter in Group A (received foam sclerotherapy) as compared to Group B (did not receive foam sclerotherapy): median 35 days, IQR 22 vs median 56 days, IQR 58; p=0.008.

**Figure II supplemental.** There was a significant improvement in Venous Clinical Severity Score (VCSS) for both groups (Group A receiving sclerotherapy and Group B not receiving sclerotherapy) with treatment p<0.0001. VCSS was assessed initially upon presentation, and after complete ulcer healing.
